# Supplementary material for: Juvenile Exposure to BPA Alters the Estrous Cycle and Differentially Increases Anxiety-like Behavior and Brain Gene Expression in Adult Male and Female Rats
Source: Toxics. 2022 Aug 30;10(9):513. doi: 10.3390/toxics10090513 (PMC9505797; doi:10.3390/toxics10090513)
Supplement: Supplementary file 1 [file toxics-10-00513-s001.zip › toxics-1831232-supplementary material.pdf]

toxics

MDPI

# Supplementary Materials: Juvenile Exposure to BPA Alters the Estrous Cycle and Differentially Increases Anxiety-like Behavior and Brain Gene Expression in Adult Male and Female Rats

**Laura Yesenia Castillo, Jorge Ríos-Carrillo, Juan Carlos González-Orozco, Ignacio Camacho-Arroyo, Jean-Pascal Morin, Rossana C. Zepeda and Gabriel Roldán-Roldán**

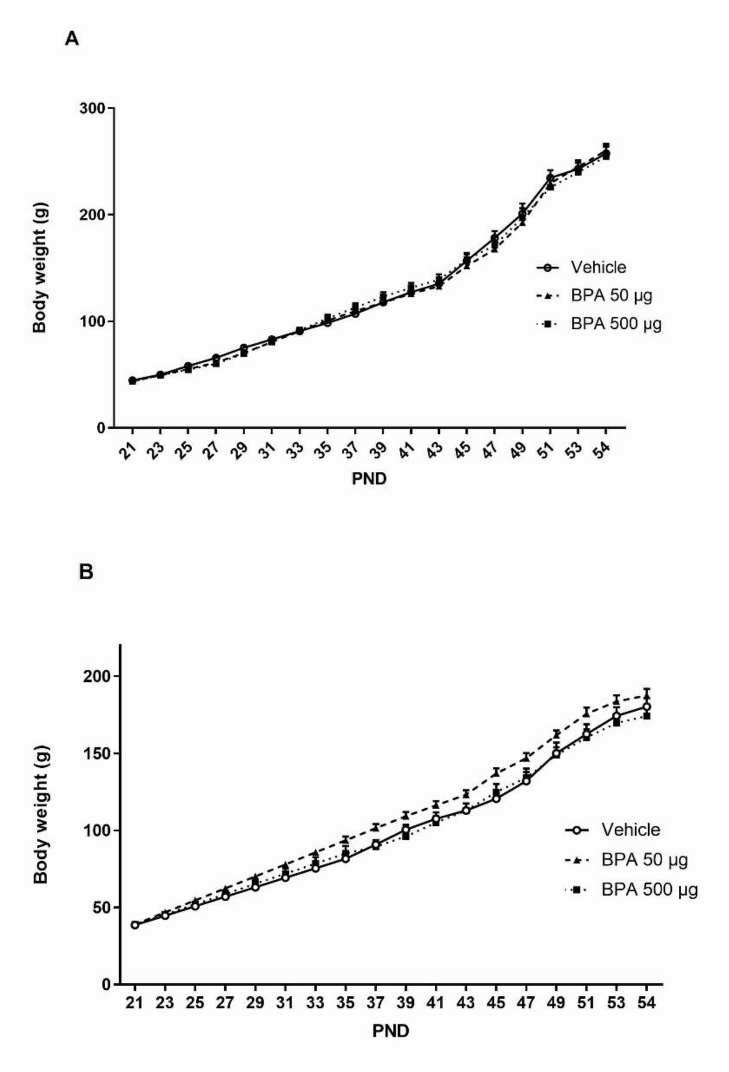


**Figure S1.** Average body weight as a function of age (in PNDs) in males (A) and females (B). The mean weight ±SEM is graphed for each time point.

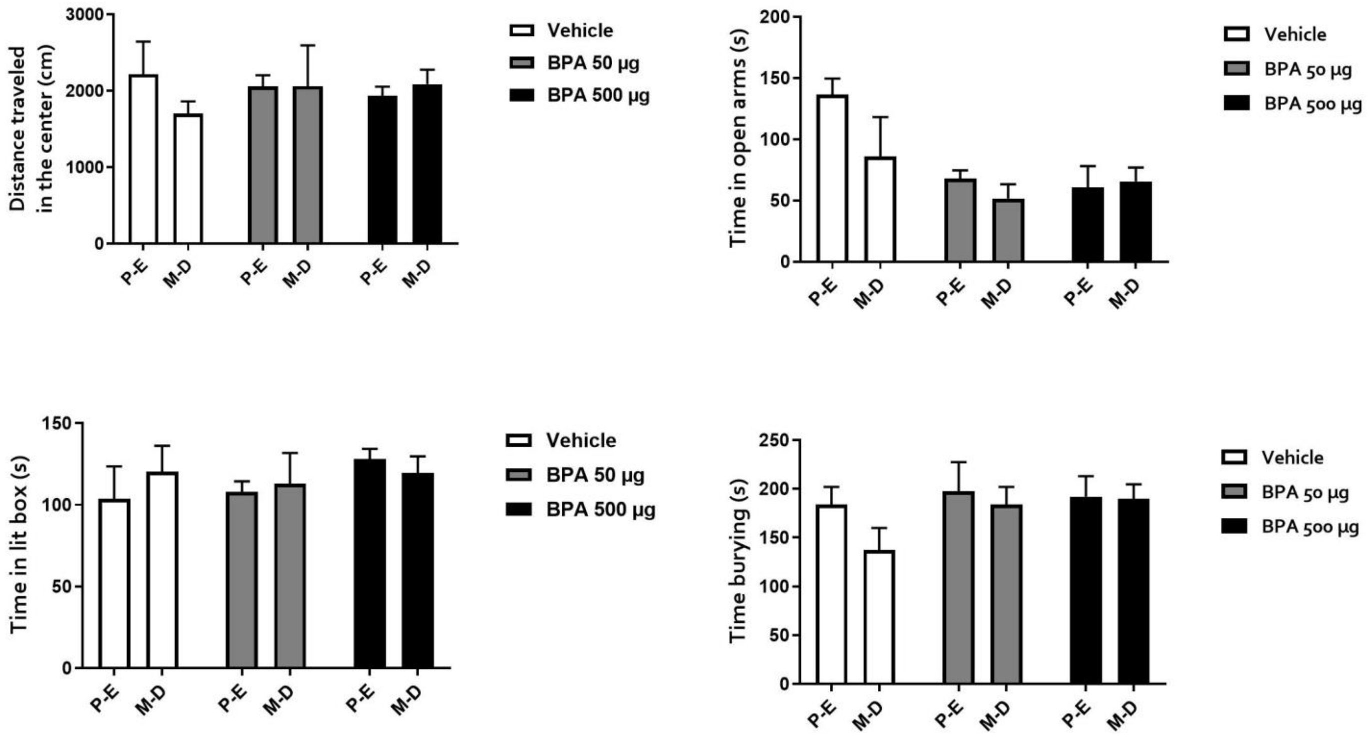


**Figure S2.** Anxiety-like behaviors in females in the four tasks evaluated in the present study, as a function of estrous cycle phase. P-E: Proestrous-Estrous; M-D: Metestrous-Diestrous. A) OF; B) EPM; C) LDBT; D) DBT. Bars represent mean ±SEM.

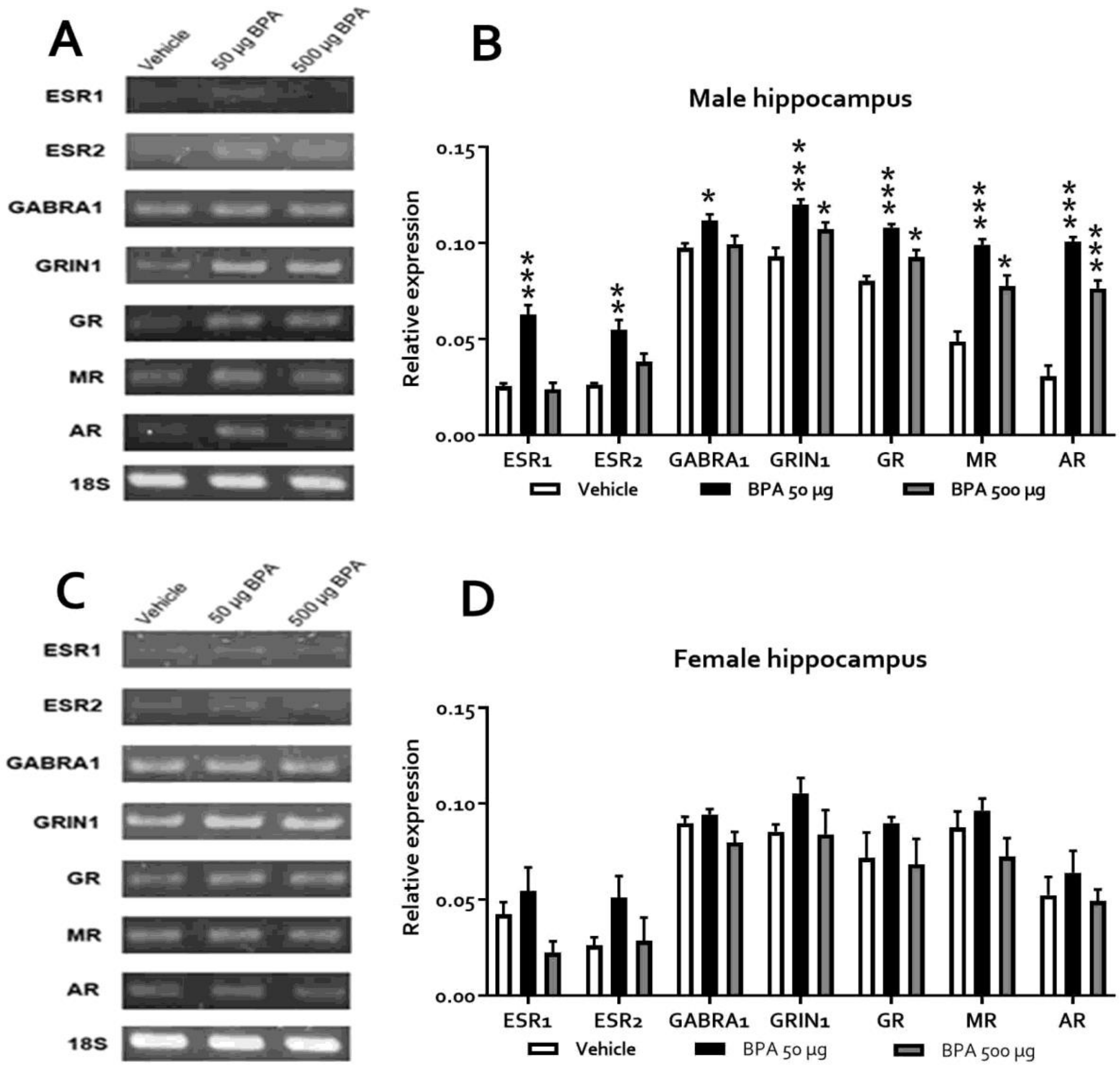


**Figure S3.** BPA affects gene´s expression associated with the anxiety response in the male and female hippocampus. A) Representative image of end point-PCR products on an agarose gel showing the expression levels of genes Estrogen Receptor 1 (*ESR1* and *ESR2*), alfa1 subunit of $GABA_A$ receptor *GABRA1*, Glutamate Ionotropic Receptor NMDA Type Subunit 1 (*GRIN1*), Glucocorticoid Receptor (*GR*), Mineralocortocoid (*MR*) and Androgene receptor (*AR*) in the male hippocampus. The label on the left side of each row corresponds to the specific studied genes, while the upper label corresponds to the employed BPA treatments. B) The graph shows the expression levels of the studied genes in the male hippocampus obtained from densitometric analysis of the agarose gels. C) Representative image of end point-PCR products in an agarose gel showing the expression levels of the studied genes in the female hippocampus. D) The graph shows the expression levels of the studied genes in the female hippocampus obtained from densitometric analysis of the agarose gels. The results are represented as the relative expression with respect to 18S. Each bar represents the $\overline{X}$ ± SEM, ($n$ = 3–5/group). *$p < 0.05$, **$p < 0.001$, and ***$p < 0.0001$ *vs*. male vehicle group.

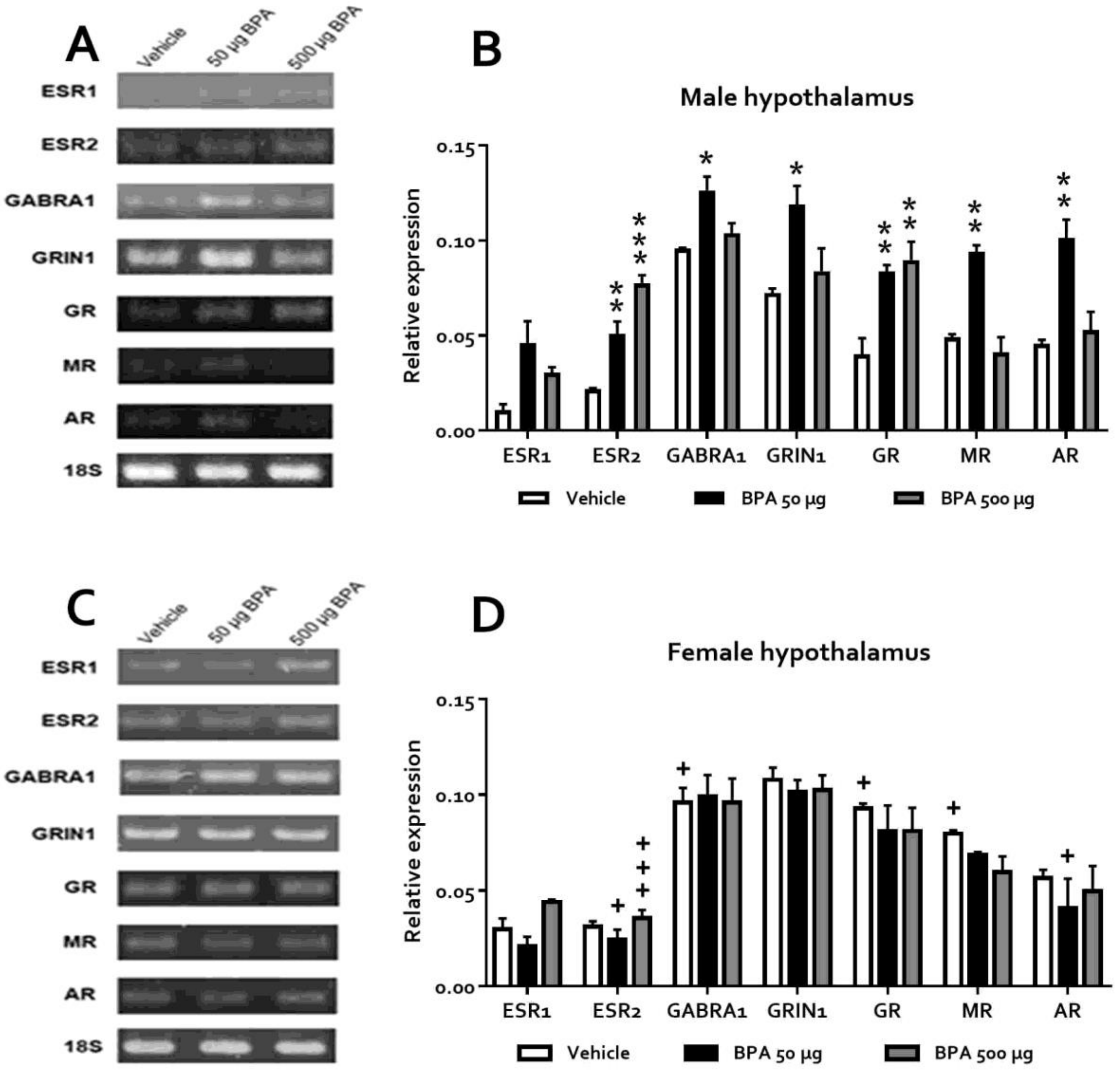


**Figure S4.** BPA effects on the expression of genes associated with the anxiety response in male and female hypothalamus. A) A representative image of end point-PCR products on an agarose gel shows the expression levels of the studied genes ESR1 and 2, GABRA1, GRIN1, GR, MR and AR in the male hypothalamus. The label on the left side of each row corresponds to the specific studied genes, while the upper label corresponds to the employed BPA treatments. B) The graph shows the expression levels of the studied genes in the male hypothalamus obtained from densitometric analysis of the agarose gels. C) Representative image of end point-PCR products in an agarose gel showing the expression levels of the studied genes in the female hypothalamus. D) The graph shows the expression levels of the studied genes in the female hypothalamus obtained from densitometric analysis of the agarose gels. The results are represented as the relative expression with respect to 18S. Each bar represents the $\bar{X}$ ± SEM, ($n$ = 3–5/group). *$p < 0.05$, **$p < 0.001$ and ***$p < 0.0001$ *vs.* male vehicle group.

**Table S1. Monitoring of the estrous cycle of DPN 28-54.**

| | | | | | | | | | | | | | | | | | | | | | | | | | Behavioral tests | | | |
|---|---|---|---|---|---|---|---|---|---|---|---|---|---|---|---|---|---|---|---|---|---|---|---|---|---|---|---|---|
| | | | | | | | | | | | | | | | | | | | | | | | | | OFT | EPM | DLBT | DBT |
| | DPN | 28 | 29 | 30 | 31 | 32 | 33 | 34 | 35 | 36 | 37 | 38 | 39 | 40 | 41 | 42 | 43 | 44 | 45 | 46 | 47 | 48 | 49 | 50 | 51 | 52 | 53 | 54 |
| Vehicle | R1 | A | Dp | P | Em | M | D | Dp | E | eM | D | D | Pe | E | eM | D | D | dE | eM | D | D | Pe | E | eM | mD | Pe | E | M |
| | R2 | A | A | Dp | Pe | E | M | D | Pe | E | Md | D | dP | E | Md | D | Dp | E | Md | Dp | E | eM | D | D | dP | E | eM | D |
| | R3 | A | Dp | dP | P | Pe | E | M | D | Pe | E | Md | D | P | E | eM | D | Dp | E | eM | D | Pe | E | D | De | E | M | mD |
| | R4 | A | Dp | P | D | P | Pe | E | M | D | dP | E | eM | mD | D | Pe | E | Md | D | Dp | E | Md | D | Dp | E | M | mD | dP |
| | R5 | A | Dp | Pe | E | M | D | dE | D | Dp | E | Md | D | Dp | E | eM | D | Dp | E | eM | mD | Pe | E | mD | D | Pe | E | Md |
| | R6 | A | A | Dp | Pe | E | mD | Dp | E | Md | D | dP | E | Md | D | Dp | E | eM | D | dP | E | Md | D | Dp | E | eM | D | Dp |
| | R7 | A | A | Dp | D | P | E | M | D | pE | M | D | Pe | E | M | mD | dP | E | Md | D | dP | E | Md | D | Dp | E | Md | D |
| | R8 | A | A | A | P | P | E | Em | Md | D | dP | E | M | mD | pE | Md | D | Dp | E | eM | D | Dp | E | eM | D | D | pE | D |
| | R9 | A | A | Dp | Dp | Dp | dE | eM | mD | D | Pe | E | Md | D | Pe | E | Md | Dp | P | E | eM | D | Pe | E | Md | D | dP | E |
| BPA 50 µg/kg | R1 | A | Pe | mD | pE | P | pE | E | Md | pE | Md | E | Md | Dp | E | mD | E | Md | D | dP | dE | E | Md | Em | dE | mD | E | D |
| | R2 | A | Pe | mD | pE | E | Md | De | E | M | mD | E | Md | De | Ed | E | Md | De | Em | dE | mD | pE | E | Md | D | Em | mD | dP |
| | R3 | A | Pe | E | Ed | Md | pE | D | E | P | Em | D | E | P | E | D | Em | De | E | mD | D | pE | Md | dE | E | D | Em | De |
| | R4 | A | Pe | Em | D | pE | eD | Pe | E | M | mD | D | pE | eD | Em | E | Md | D | E | mD | dE | E | eM | E | M | E | Md | D |
| | R5 | dE | Pe | E | Em | Pe | Em | Dp | E | Md | Pe | Ed | Dp | E | eD | E | dE | D | E | mD | dE | E | D | dE | M | E | dE | D |
| | R6 | A | A | D | dE | D | D | dE | eM | M | mD | dE | D | pE | eM | D | E | mD | Em | D | E | mD | dE | D | pE | mD | Em | Md |
| | R7 | A | Dp | pE | mD | pE | D | dE | M | De | Ed | M | E | Md | D | Em | D | E | mD | E | Md | De | E | Md | De | E | Md | dE |
| | R8 | dE | Md | pE | dE | Me | Dp | Ed | D | Em | Md | Dp | E | Dp | Ed | E | mD | De | Em | De | Em | D | Em | dP | E | mD | De | Em |
| | R9 | A | Pe | Md | Em | De | E | Em | De | Pe | Ed | mD | De | Em | D | pE | Md | De | Em | D | E | Em | De | D | pE | mD | De | Em |
| BPA 500 µg/kg | R1 | A | D | dE | D | Em | M | D | dE | mD | dE | mD | dE | Md | Em | Md | eD | Em | mD | E | mD | dE | mD | dE | E | M | D | dE |
| | R2 | Dp | Pe | E | D | pE | mD | De | Md | De | dE | mD | dE | eM | D | dE | mD | dE | Md | De | E | mD | De | E | Em | De | E | D |
| | R3 | A | pE | M | mD | pE | eD | De | E | eD | Pe | Em | Me | D | Ed | E | De | Ed | E | De | E | eD | dE | Md | E | eD | Pe | dE |
| | R4 | Dp | Pe | Em | eD | Pe | Ed | D | dE | E | Md | E | De | P | Ed | Em | De | E | Md | De | D | dE | E | mD | dE | Md | E | Md |
| | R5 | A | Pe | Em | eD | pE | eM | E | Md | De | Ed | Pe | E | eM | dE | Md | De | E | Md | De | E | eM | De | Md | E | Md | De | mD |
| | R6 | Dp | D | D | Ed | Pe | De | E | eM | dE | Md | Em | De | E | eD | E | mD | dE | Md | De | E | eM | dE | D | E | eM | dE | D |
| | R7 | mD | dP | pE | eD | Pe | Ed | Md | De | E | E | eM | De | E | D | E | eM | D | pE | mD | E | E | mD | dE | Md | De | E | eM |
| | R8 | | Em | De | Em | mD | Ed | M | E | mD | Md | Ed | mD | dP | E | De | Md | E | mD | E | eD | eM | E | Md | dE | Em | Md | D |
| | R9 | | Em | Md | E | eD | De | Em | mD | E | mD | Ed | mD | dP | E | E | Md | De | Em | mD | E | Ed | D | dE | E | Md | dE | mD |
| | R10 | mD | Pe | E | mD | pE | Md | De | Ed | Dp | E | mD | Ed | D | dE | Md | De | E | mD | dP | E | eD | De | E | E | Md | D | Dp |
| | R11 | A | D | dP | E | mD | dE | eD | dP | E | Md | D | Em | D | mD | dE | eM | D | pE | mD | E | M | Dp | Em | D | dE | mD | E |
| | R12 | pE | mD | E | mD | E | Md | Dp | E | mD | Dp | Em | D | dE | mD | E | eM | D | dP | E | eM | D | Pe | Ed | De | E | D | Dp |
| | R13 | Dp | E | mD | E | Md | Dp | E | mD | Pe | E | Md | Dp | E | mD | dP | De | pE | eD | E | Md | Dp | E | eM | D | Pe | E | eD |
| | R14 | Dp | E | D | dE | Md | Dp | E | mD | E | mD | dP | E | eM | D | pE | mD | pE | Md | E | Dp | Em | D | dE | eM | D | dP | E |
| | R15 | Dp | Em | D | dE | eM | dE | dP | E | eD | Em | eD | dP | E | Md | dE | D | Dp | E | eD | Pe | E | De | Em | D | De | E | pE |
| | R16 | Md | D | E | Em | D | De | E | mD | Dp | E | eM | D | pE | mD | Em | D | dE | Em | D | Em | D | dP | E | Md | Dp | Em | dE |
| | R17 | Dp | E | Md | D | pE | Em | D | E | M | dP | E | E | eM | De | mD | E | eM | E | eD | dP | E | D | E | mE | D | Md | dE |

A, anestrous; P, proestrus; E, estrus; M, metaestrus; D, diestrus, Lowercase letter indicates the stage of lowest prevalence, capital letter indicates the stage of highest prevalence. The black shaded boxes indicate the first observed Estrus, the gray shaded boxes indicate the first proestrus observed (P or p) in some cases it occurred in intermediate stages, therefore we start from the first well-defined Estrus. DPN 51-54 behavioral tests were performed.

**Table S2. List of employed primers.**

| *Primer (3´→5´)* | *Sequence* | *Tm* | *Amplicon size* |
|---|---|---|---|
| AR Forward<br>AR Reverse | CTTATGGGGACATGCGTTTGG<br>GCTCCGTAGTGACAACCAGA | 60°C | 121 pb |
| ESR1 Forward<br>ESR1 Reverse | AATGTCGTGCCTCTCTATG<br>TTGTAAGGAATGTGCTGAAGT | 58°C | 147 pb |
| ESR2 Forward<br>ESR2 Reverse | GGGTGATTGCGAAGAGTGGTA<br>GCCTGACGTGAGAAAGAAGCA | 59°C | 84 pb |
| GABRA1 Forward<br>GABRA1 Reverse | TGACAGTCATTCTCTCCCAAGTC<br>TCAGAACGGTCGTCACTCC | 57°C | 87 pb |
| GRIN1 Forward<br>GRIN1 Reverse | TACTCCCAACGACCACTTCAC<br>ACTCGCATCATCTCAAACCAG | 60°C | 183 pb |
| GR Forward<br>GR Reverse | AAAGGCGATACCAGGCTTCA<br>GATCTCCAACCCAGGGCAAA | 60°C | 102 pb |
| MR Forward<br>MR Reverse | TGTCTCAGACCTTGGAGCGTT<br>TGTTCGGAATAGCACCGGAA | 60°C | 108 pb |
| 18S Forward<br>18S Reverse | AGTGAAACTGCAATGGCTC<br>CTGACCGGGTTGGTTTTGAT | 60°C | 167 pb |
